# Effects of aerobic exercise on myocardial injury, NF-B expression, glucolipid metabolism and inflammatory factors in rats with coronary heart disease

**DOI:** 10.1016/j.clinsp.2024.100386

**Published:** 2024-05-29

**Authors:** Shaowu Zhang, Guohui Yu, Maohua Ping, Qing Du, Xia Guo

**Affiliations:** Department of Rehabilitation Medicine, Wuhan Hanyang Hospital, Wuhan City, Hubei Province, China

**Keywords:** Coronary heart disease, Aerobic exercise, Glucolipid metabolism, NF-B signaling pathway, Inflammation

## Abstract

**Objective:**

To investigate the influence of aerobic exercise on myocardial injury, NF-B expression, glucolipid metabolism and inflammatory factors in rats with Coronary Heart Disease (CHD) and explore the possible causative role.

**Methods:**

45 Sprague Dawley® rats were randomized into model, control and experimental groups. A high-fat diet was adopted for generating a rat CHD model, and the experimental group was given a 4-week aerobic exercise intervention. ECG was utilized to evaluate the cardiac function of the rats; HE staining to evaluate the damage of myocardial tissue; TUNEL staining to evaluate cardiomyocyte apoptosis level; ELISA to assay the contents of inflammatory factors and glucolipid metabolism in cardiomyocytes; qPCR to assay IB- and NF-B mRNA expression; Western-blot to assay the apoptosis-related proteins and NF-B signaling pathway-related proteins expressions in myocardial tissue.

**Results:**

In contrast to the model group, aerobic exercise strongly improved the rat's cardiac function and glucolipid metabolism (p < 0.01), enhanced IL-10 content, Bcl-2/Bax level as well as IB- protein and mRNA expression (p < 0.01), and reduced myocardial injury and cardiomyocyte apoptosis, the contents of IL-6, IL-1 and TNF-, Caspase 3 level, NF-B mRNA and protein expression and p-p38 and p-STAT3 expressions (p < 0.01).

**Conclusion:**

Aerobic exercise can not only effectively reduce myocardial injury, the release of inflammatory factors and NF-B expression in CHD rats, but also improve cardiac function and glucolipid metabolism. Its mechanism is likely to be related to the inhibition of the NF-B signaling pathway.

## Highlights

• Aerobic exercise strongly improved cardiac function and glucolipid metabolism in CHD.
• Aerobic exercise enhanced IL-10 content, Bcl-2/Bax level as well as IB- expression in CHD.
• Aerobic exercise reduced myocardial injury and cardiomyocyte apoptosis in CHD.

## Introduction

Coronary Heart Disease (CHD) refers to coronary artery lumen stenosis or even blockage caused by atherosclerosis, which causes myocardial ischemia, hypoxia and even necrosis, and is also known as ischemic heart disease.^1^^,^^2^ Physiological, genetic and environmental factors that cause aberrant glucolipid metabolism and inflammatory response disorders are the dominating incentives that induce atherosclerosis and the primary pathophysiological basis of CHD.^3^^,^^4^ With the improvement in living standards, the incidence of CHD is increasing year by year, posing a huge economic burden on families and countries. Reducing the mortality and extending the survival time of CHD patients is a major challenge that cardiovascular disease researchers and clinicians urgently need to solve.^5^^,^^6^

Considerable evidence shows that vascular endothelial injury will raise the adhesion and permeability of vascular endothelium, and then multiple inflammatory factors will gather and produce an inflammatory fiber proliferation reaction, which eventually gives rise to atherosclerosis.^7^, ^8^, ^9^ Nuclear Factor Kappa B (NF-B) takes part in not only the occurrence but also the development of inflammatory response. NF-B inhibitor protein IB- can accelerate the dissociation of NF-B. The dissociated NF-B can be rapidly transferred to the nucleus, modulating the transcription and translation processes of diverse inflammatory factors and adhesion factors, and is an important cytokine that modulates the activation and cascade amplification of inflammatory factors.^10^^,^^11^ Schiffmann et al.^12^ have demonstrated that CHD patients will have apparent glucolipid metabolism disorder. Over-nutrition and sedentary behaviors will strikingly accelerate the accumulation of fat and glucose in the body, which in turn will aggravate the vascular endothelial damage of patients. Substantial evidence indicates that aerobic exercise can efficaciously regulate glucolipid metabolism in the body, reduce the activity of lipoprotein lipase, lower blood lipid and blood sugar levels, and reduce body weight.^13^^,^^14^ Ornish et al.^15^ demonstrated that long-term aerobic exercise can apparently reduce blood lipid levels and reduce vascular endothelial damage in patients with cardiovascular disease. At present, there are few studies on the impact of aerobic exercise on myocardial damage, glucolipid metabolism, and inflammation in CHD. This investigation aimed to build a rat CHD model to study the influence of aerobic exercise on myocardial damage, NF-B expression, glucolipid metabolism and inflammatory responses in CHD rats, as well as to further explore its possible causative role.

## Materials and methods

### Experimental animals and groups

Forty-five healthy Sprague Dawley® (SD) rats (males) were procured from the Experimental Animal Center of Harbin Medical University, weighing 220‒250g. Batch number: SCXK (Black) 2018-001. The feeding environment was SPF grade, the humidity was 45%±5%, the temperature was 21±3℃, and the circadian rhythm was followed. The rats adapted to the environment for 7 days and were permitted *ad libitum* access for food and water. Subsequently, rats were randomly assigned into the model group, the control group, and the experimental group, with n = 15 rats in each group. Each rat was uniquely labeled and weighed before the experiments. The rats were fed with normal feed in the control group, whereas the rats were fed with a high-fat diet in the experimental group and the model group to construct a CHD model. The experimental group performed moderate-intensity treadmill exercises after successful CHD modeling.

This experimental protocol was authorized by the Experimental Animal Ethics Committee of Wuhan Hanyang Hospital. All experimental operations in this protocol were carried out in strict accordance with the relevant provisions of the NIH guidelines for experiments in animals.

### Reagents and instrumentation

High-fat feed (Beijing Keao Xieli Feed Co., Ltd., China); pentobarbital sodium (Sigma); ethanol, formalin and ammonia (Tianjin Kermel Chemical Reagent Co., Ltd., China); RIPA lysate, TUNEL staining reagent kit (Beyotime); TNF-, IL-10, IL-1 and IL-6 ELISA kits (Boster); qPCR kit (Vazyme Biotech Co., Ltd, Nanjing, China); Caspase3 monoclonal antibody, Bcl-2 monoclonal antibody, NF-B monoclonal antibody, IB- monoclonal antibody, p-p38 monoclonal antibody, p38 monoclonal antibody, p-STAT3 monoclonal antibody, STAT3 monoclonal antibody, GAPDH monoclonal antibody, goat anti-rabbit IgG-horseradish peroxidase (HRP) (Cell Signaling Tech, USA); BL-420E+ biological signal acquisition (Mindware, USA); microplate reader (Bio-Tek, USA); fluorescent quantitative PCR instrument (Bio-Rad, USA); automatic biochemical analyzer (Roche, Swiss); confocal fluorescence microscope (Nikon, Japan).

### Rat CHD model and aerobic exercise intervention

Establishment of rat CHD model: A high-fat diet (87.3% basic feed, 0.5% sodium cholate, 0.2% propylthiouracil, 2% cholesterol as well as 10% lard) feeding method was adopted for constructing a rat CHD model: Gavage once a day for 6-weeks at a dose of 10 mL/kg/d; intraperitoneal injection of 30 U/kg pituitrin for 3 consecutive days before the last gavage; on the last day, record Left Ventricular Internal Diameter end systole (LVIDs), Left Ventricular Internal Diameter end diastole (LVIDd), Left Ventricular Fractional Shortening (LVFS) and Left Ventricular Ejection Fraction (LVEF) of each group of rats adopting BL-420E+ biosignals acquisition device; evaluate the cardiac function of rats and determine whether the rat CHD model is successfully constructed.

Aerobic exercise intervention: The rats in the experimental group first performed 3-days of adaptive training on the treadmill, and then 4-weeks of moderate-intensity aerobic exercise, once a day. Week 1: Speed was 15 meters/min, exercise time was 30 min; Week 2: Speed was 15 meters/min, exercise time was 60 min; Week 3: Speed was 20 meters/min, exercise time was 60 min; Week 4: Speed was 20 m/min, exercise time was 90 min.

### ECG to detect cardiac function in rats

On the second day after the aerobic exercise intervention, a BL-420E+ biological signal acquisition and analysis device (Mindware, USA) was employed for recording the LVIDd, LVIDs, LVEF, and LVFS of each group of rats.

### HE-staining to assay the morphological changes of rat myocardium

On the second day after the end of aerobic exercise intervention, HE-staining was used for detecting morphological changes in myocardial tissue in each group of rats: after anesthetizing the rats with pentobarbital sodium, the myocardial tissue was separated immediately, successively dehydrated with 50%, 60%, 70%, 80%, 90%, 95% and 100% alcohol after fixation, and transparent in xylene to prepare paraffin sections. Paraffin sections were cut into slices with a thickness of 10 μm in a microtome, placed on coated slides, dried and then dewaxed. The slices were successively soaked in 90%, 80%, 70%, 60% and 50% alcohol for 2 min, soaked in water for 30s, stained with hematoxylin for 6 min, washed with water, successively soaked in hydrochloric acid, alcohol and ammonia for 30s each time, washed with water, dyed in eosin for 30s, successively soaked in 50%, 60%, 70%, 80% and 90% alcohol for 5 min, fully transparent, and finally performed sealing applying neutral resin to observe and photograph taking advantage of confocal fluorescence microscope (Nikon, Japan).

### TUNEL staining to assay the apoptosis of rat cardiomyocytes

On the second day after the aerobic exercise intervention, the TUNEL staining method was adopted for measuring the apoptosis of rat cardiomyocytes: paraffin sections of rat myocardial tissue in each group were dewaxed into water, treated with 3% hydrogen peroxide lasting 10 min, and washed three times with PBS. The procedures were conducted following the instructions of the TUNEL kit (Beyotime Biotechnology, China). Observation image capture was undertaken using the confocal fluorescence microscope, and TUNEL-positive cells in cardiomyocytes were recorded to calculate the cardiomyocyte apoptosis levels.

### ELISA to assay fasting blood lipid and glucose levels in rats

After the aerobic exercise intervention, rats were fasted for 12h. An Oral Glucose Tolerance Test (OGTT) was administered: rats in each group were given 50% glucose with equal weight ratio by gavage; 0 min, 15 min after gavage, blood was collected through the tail vein at 30 min, 60 min and 12 min to measure glucose concentration and calculate the glucose tolerance of mice in each group. After the end of the OGTT, anesthetization of rats was performed employing pentobarbital sodium, and collection of blood was completed from the inferior vena cava. After standing for 15 minutes, serum was harvested by centrifuging at 4°C and 3000 rpm for 10 minutes. Concentrations of High-Density Lipoprotein Cholesterol (HDL-C), Low-Density Lipoprotein Cholesterol (LDL-C), Triglyceride (TG) as well as Total Cholesterol (TC) in were assayed using the hospital's automatic biochemical analyzer (Roche, Basel).

### ELISA to assay the contents of inflammatory factors in rat myocardium

The rats were anesthetized with pentobarbital sodium (Sigma Aldrich, St. Louis, MO) on the second day after the aerobic exercise intervention. The rat myocardial tissue was isolated and homogenized. After 15 minutes, supernatant was harvested after centrifuging at 4°C and 12000 rpm for 10 minutes. Concentrations of inflammatory factors IL-1, TNF-, IL-10, and IL-6 in the myocardial tissue of rats in each group were assayed using commercially available ELISA kits (Boster Bio, Pleasanton, CA), according to kit instructions. A microplate reader was used for detecting the absorbance value (Bio-Tek Instruments, Winooski, VT).

### qPCR to assay related gene expression levels

A portion of the rat myocardial tissue was isolated for RNA analysis. Trizol reagent was added to extract the total RNA. Total RNA concentration of each group of samples was measured and the reverse transcription reaction was undertaken. The reaction conditions were set as 37°C for 15 min, 85°C for 5 sec. Total RNA was reverse transcribed to cDNA and saved for future use. qPCR was then undertaken according to the kit instructions (Vazyme Biotech Co., Ltd, Nanjing, China), and the reaction conditions were set to 95°C 30 sec, 95°C 5 sec, 60°C 35 sec, 40 cycles. Adopting GAPDH as an internal reference, 2^−ΔΔCt^ was used to calculate NF-B, IL-1, TNF-, IL-10, and IL-6 expression levels in each group of samples. The primers were produced by Thermo Fisher Scientific Co., Ltd. Shanghai (Shanghai, China) Primer sequences are given in Table 1.

**Table 1:** PCR primers.

	Sequence
**NF-B**	Forward primer: 5’-GGCTGAAATGCTGTACGTCGGT-3’
	Reverse primer: 5’-CCGGTAGGTCATGGGTCACTCT-3’
**IB-**	Forward primer: 5’-TTGAGTCGTAGACCTGACTGC-3’
	Reverse primer: 5’-TATGGTTGGGTCGTGCTGTCTC-3’
**GAPDH**	Forward primer: 5’-ATTGGAACGATACAGAGATT-3’
	Reverse primer: 5’-GGAACGCTTCACGAATTTG-3’

### Western blot to assay related protein expression level

Rat myocardial tissue was separated for western blot analysis. RIPA lysate was added, and 1% phosphatase inhibitor and protease inhibitor were added on the basis of the volume ratio. The homogenate was sonicated until there was no visible tissue and centrifuged at 4°C, 12000 rpm for 10 minutes. The supernatant was recovered, and the BCA protein quantification kit was used to assay the concentration of each group of proteins. After adding an equal concentration of loading buffer protein was transferred to the PVDF membrane through electrophoresis, newly configured 5% skimmed milk powder was added for 1 hour, protein band was recovered and incubation was undertaken with the corresponding primary antibodies Caspase 3, Bcl-2, Bax, NF-B, IB-, p-p38, p38, p-STAT3, STAT3 and GAPDH (monoclonal antibodies obtained from Cell Signaling Tech, Danvers, MA) (1:1000) overnight. After washing 3 times with TBST, incubation with goat anti-rabbit IgG-HRP (1:5000) was performed for 2 hours under room temperature conditions. Following 3 further washes with TBST, freshly prepared luminescent solution was added for development and exposure. Image J software (NIH, Bethesda, MD) was employed for analyzing the expression level of each protein after scanning.

### Statistical analysis

Data are represented as mean ± standard deviation. Data processing was analyzed applying SPSS software version 22.0 (IBM, Chicago, IL). Comparison between groups was completed by one-way ANOVA. If the variances were homogeneous, the Bonferroni method was utilized for pairwise comparison; in the case of non-homogeneity in variances, the Welch method was utilized. A p < 0.05 for group differences was taken as statistically significant.

## Results

### Influence of aerobic exercise on rat cardiac function

**Fig. 1:**
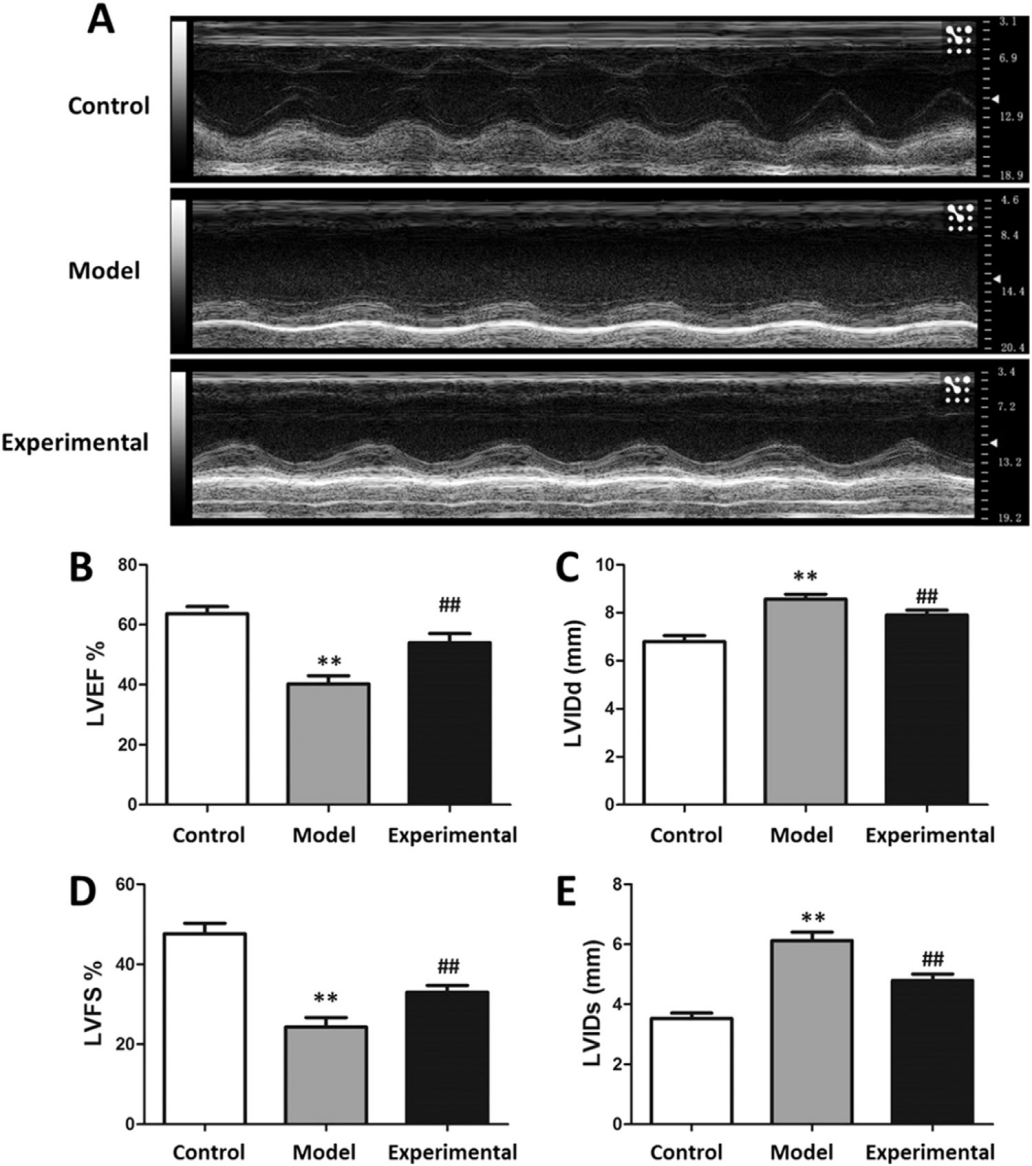
Evaluation the rat cardiac function. (A) LVEF%, (B) LVIDd, (C) LVFS%, (E) LVIDs. In contrast to control group and experimental group, LVEF% and LVFS% were lower, whereas the LVIDd and LVIDs were higher in model group. ^⁎⁎^p < 0.01 vs. control group, ^##^ p < 0.01 vs. model group.

The results of cardiac function testing are presented in Fig. 1. In contrast to the control group, the LVEF% and LVFS% of the model group were significantly reduced (p < 0.01), whereas LVIDd and LVIDs were increased (p < 0.01), suggesting that the aerobic exercise intervention can not only raise the LVEF% and LVFS%, but also reduce the LVIDs and LVIDd of rats.

### Influence of aerobic exercise on rat myocardial tissue morphology

**Fig. 2:**
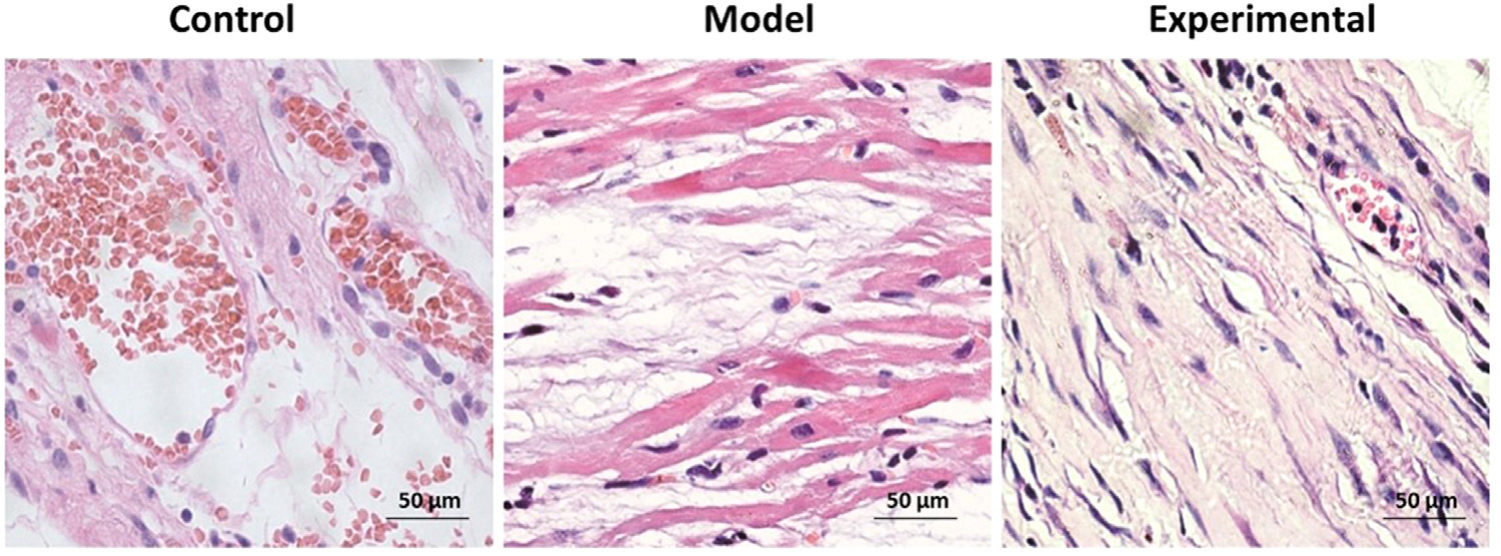
HE staining to assay the morphological changes of rat myocardium. Scale bar = 500 μm.

The results of the H&E staining are shown in Fig. 2. In contrast to the control group, widened myocardial fiber gaps and disordered and ruptured myocardial fiber arrangements were found in the model group. Moreover, inflammatory cell infiltration was observed in a large number of cells. With aerobic exercise intervention in the experimental group, shortened myocardial fiber gap, neatly arranged fiber and reduced inflammatory cell infiltration as well as lesser myocardial fiber damage was found.

### Influence of aerobic exercise on rat cardiomyocyte apoptosis

**Fig. 3:**
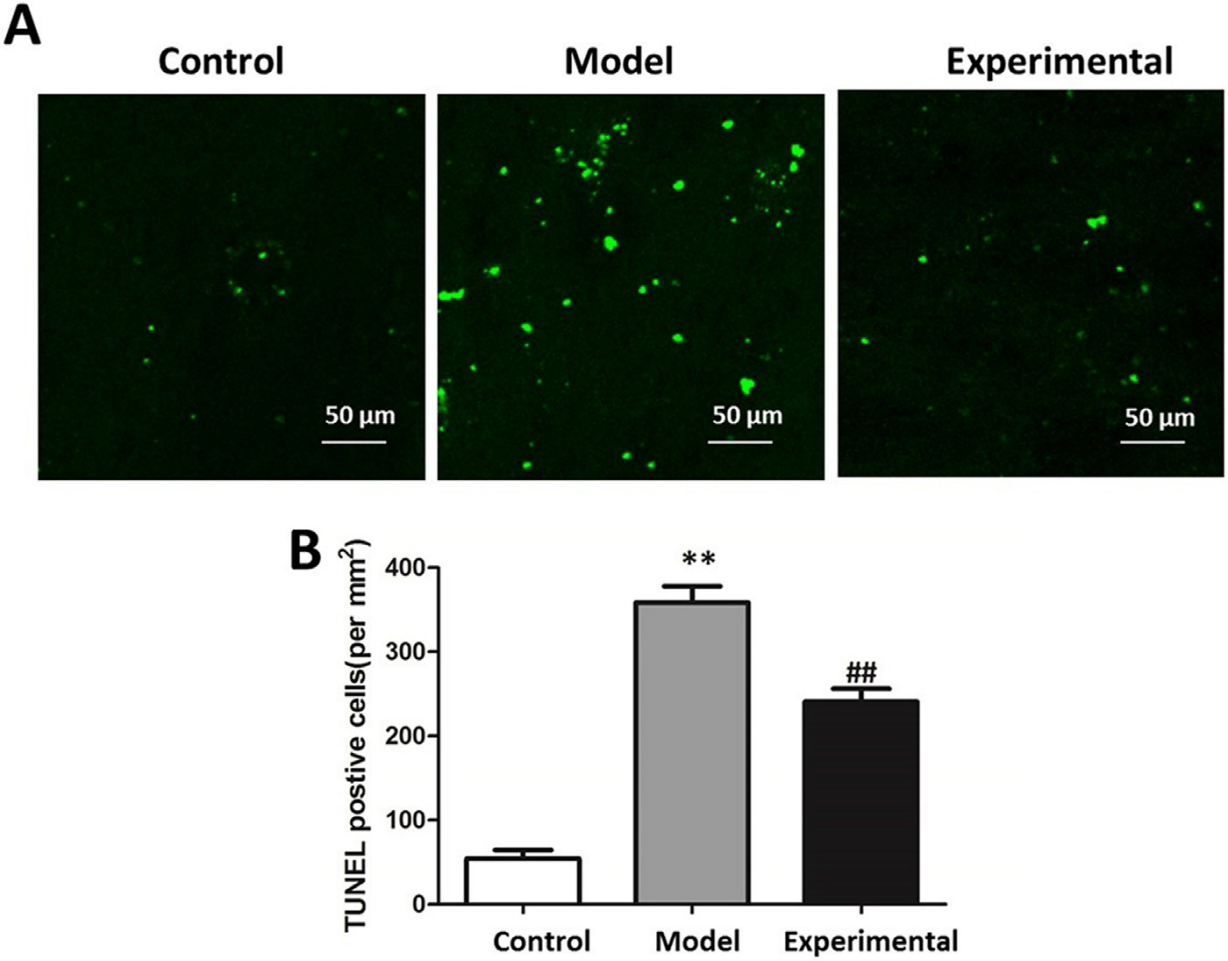
The cardiomyocyte apoptosis level in rats. (A) TUNEL staining chart. (B) Statistical chart of apoptotic cells. Scale bar = 50 μm. In contrast to control group and experimental group, cardiomyocyte apoptosis levels of model group were higher. ^⁎⁎^p < 0.01 vs. control group, ^##^ p < 0.01 vs. model group.

The results of TUNEL staining are presented in Fig. 3. In contrast to the control group, cardiomyocyte apoptosis level was significantly increased in the model group (p < 0.01), indicating that aerobic exercise intervention is capable of reducing cardiomyocyte apoptosis (p < 0.01).

### Influence of aerobic exercise on rat glucolipid metabolism

**Fig. 4:**
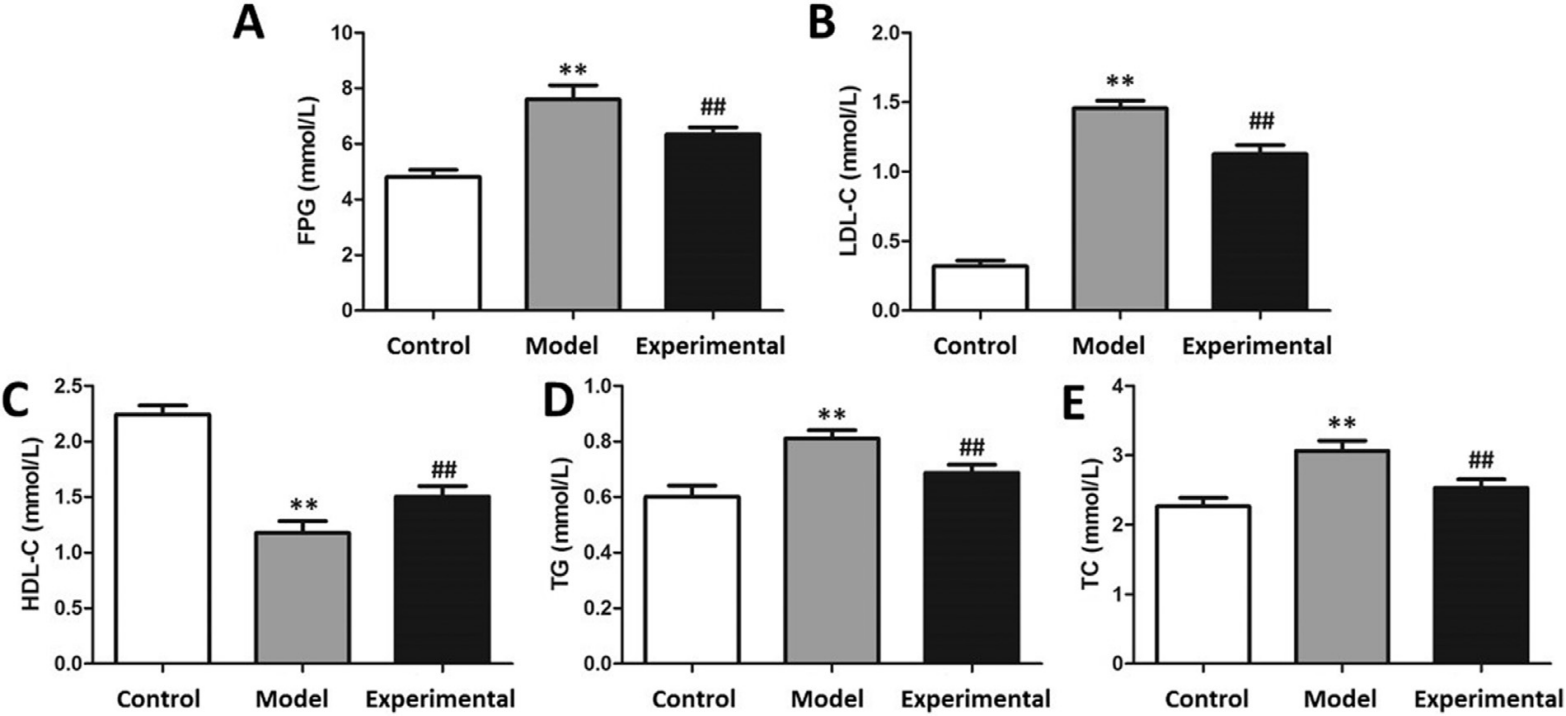
The glucose tolerance and blood lipid levels in rats. (A) FPG content. (B) LDL-C content. (C) HDL-C content. (D) TG content. (E) TC content. In contrast to control group and experimental group, the contents of FPG, LDL-C, TG and TC in model group were significantly increased, whereas HDL-C content was decreased. ^⁎⁎^p < 0.01 vs. control group, ^##^p < 0.01 vs. model group.

After the aerobic exercise intervention, the glucose tolerance and blood lipid levels of each group of rats were assayed. The results are presented in Fig. 4. In contrast to the control group, plasma glucose, LDL-C, TG and TC concentrations were markedly increased in the model group (p < 0.01), whereas HDL-C content was markedly reduced (p < 0.01). implying that aerobic exercise intervention is capable of improving glucolipid metabolism in this setting.

### Influence of aerobic exercise on inflammatory factors in rat myocardium

**Fig. 5:**
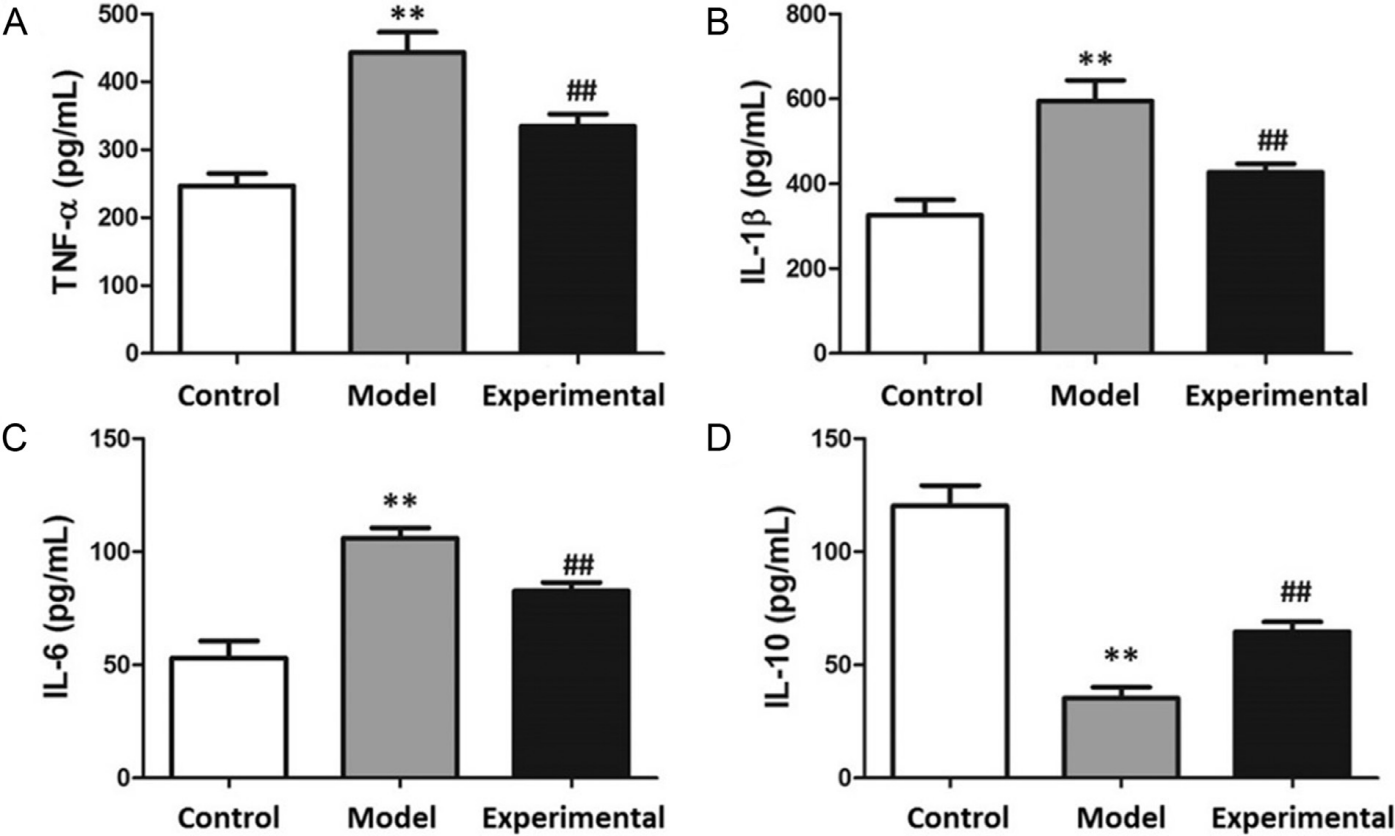
Contents of inflammatory factors in rat myocardium. (A) TNF-. (B) IL-1. (C) IL-6. (D) IL-10. In contrast to control group and experimental group, TNF-, IL-6 and IL-1 contents were increased, whereas IL-10 content was decreased in model group. ^⁎⁎^p < 0.01 vs. control group, ^##^ p < 0.01 vs. model group.

The results of the myocardial inflammatory marker analysis are presented in Fig. 5. In contrast to the control group, TNF-, IL-1, and IL-6 contents were notably increased in the model group (p < 0.01), whereas IL-10 content was reduced (p < 0.01).

### Influence of aerobic exercise on the expression of apoptotic proteins in rat myocardium

**Fig. 6:**
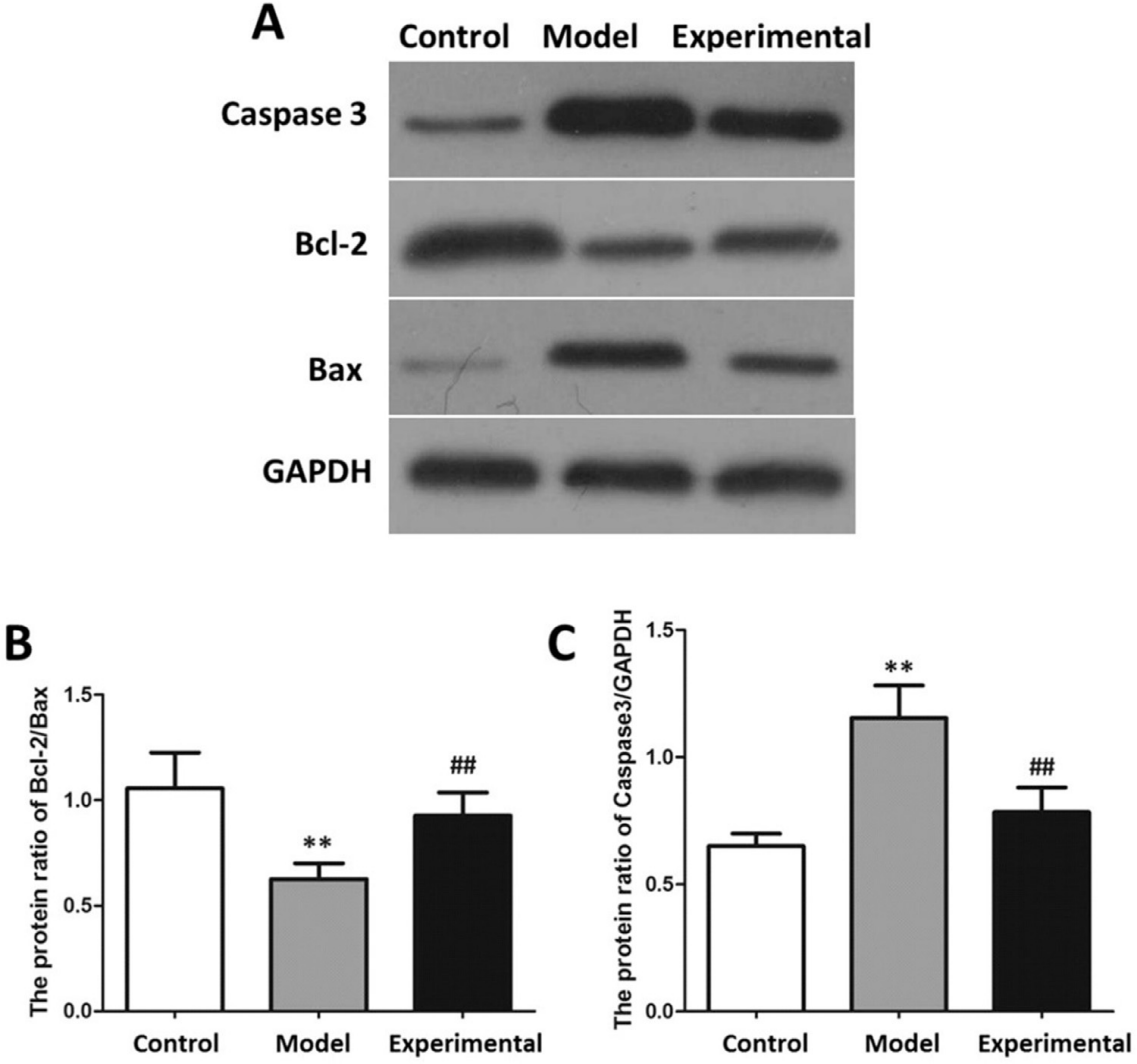
Western blot to assay apoptotic protein expression in rat myocardium. (A) protein banding. (B) Caspase 3 expression. (C) Bcl-2/Bax. In contrast to control group and experimental group, Bcl-2/Bax protein expression in model group was decreased, while Caspase 3 protein expression was increased. ^⁎⁎^p < 0.01 vs. control group, ^##^p < 0.01 vs. model group.

The results of the apoptotic protein analysis are shown in Fig. 6. In contrast to the control group, Caspase 3 expression of the myocardial tissue was increased in the model group (p < 0.01), while Bcl-2/Bax was reduced (p < 0.01).

### Influence of aerobic exercise on NF-B and IB- mRNA expression in rats

**Fig. 7:**
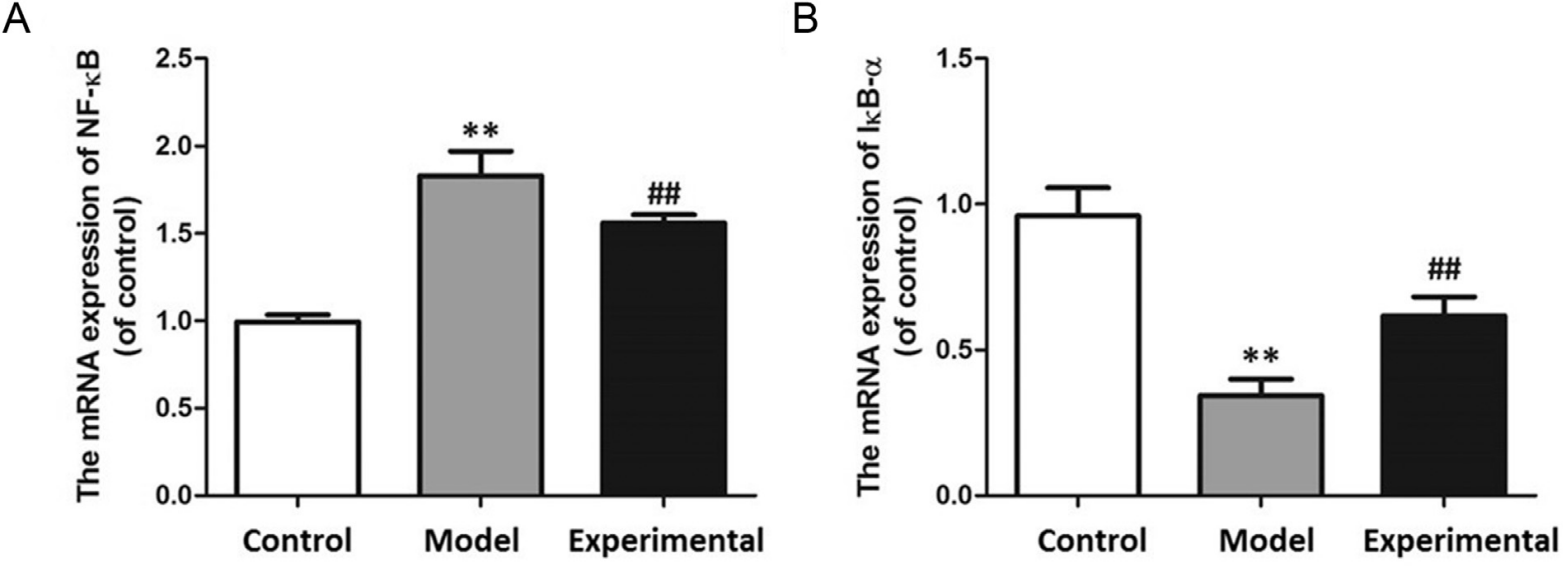
qPCR to assay mRNA expression in rat myocardium. (A) NF-B mRNA expression. (B) IB- mRNA expression. In contrast to control group and experimental group, NF-B mRNA expression in model group was increased, while IB- mRNA expression was decreased. ^⁎⁎^p < 0.01 vs. control group, ^##^p < 0.01 vs. model group.

The results pf the qPCR are shown in Fig. 7. Compared with the control group, NF-B mRNA level increased (p < 0.01), while IB- expression level decreased in rat myocardial tissues of the model group (p < 0.01).

### Influence of aerobic exercise on NF-B signaling pathway in rat myocardium

**Fig. 8:**
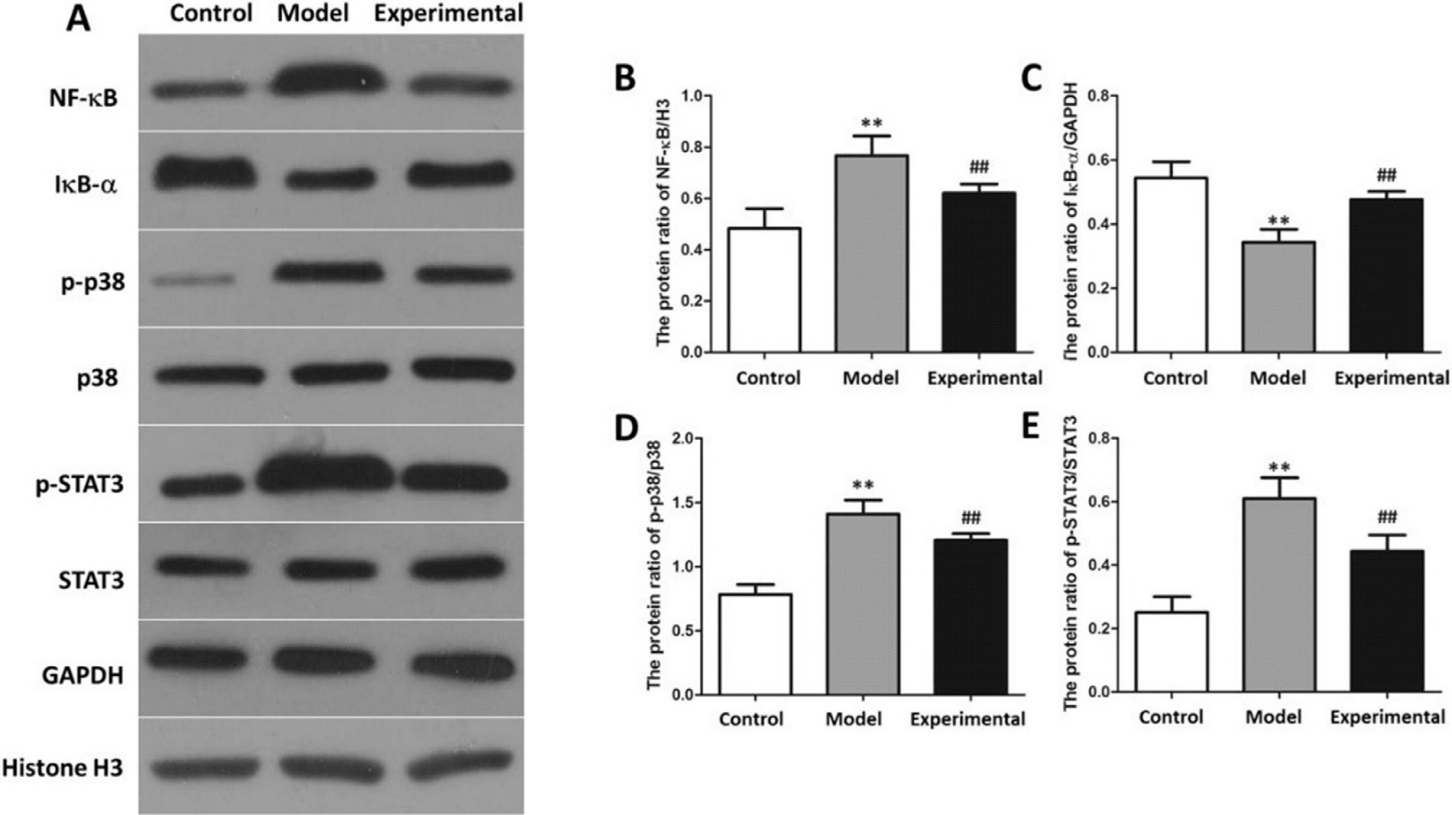
Western-blot to assay NF-B protein expression in rat myocardium. (A) protein banding. (B) NF-B expression. (C) IB- expression. (D) p-p38 expression. (E) p-STAT3 expression. In contrast to control group and experimental group, NF-B, p-p38 and p-STAT3 protein expressions in model group were increased, whereas IB- protein expression was reduced. ^⁎⁎^p < 0.01 vs. control group, ^##^p < 0.01 vs. model group.

NF-B signaling pathway-related protein expressions in the rat myocardial tissue is presented in Fig. 8. In contrast to the control group, the NF-B, p-p38 as well as p-STAT3 levels in the rat myocardial tissue of the model group were increased, whereas IB- expression levels were reduced (p < 0.01).

## Discussion

Coronary heart disease is one of the most ubiquitous cardiovascular system diseases, with the characteristics of high morbidity, high disability, and high mortality. The prognosis of CHD patients is poor and its complications are a major cause of death.^16^, ^17^, ^18^ Excess nutrition and insufficient exercise can increase body fat accumulation, glucose levels, and body weight, as well as induce various cardiovascular diseases.^19^ Substantial clinical evidence shows that aerobic exercise can effectively improve glucolipid metabolism and glucose and lipid balance and is the most common intervention for cardiovascular diseases and metabolic diseases.^20^^,^^21^ A high-fat diet feeding method to create a murine CHD model is currently recognized as an effective method, of which the success rate can reach more than 75%, Rats are highly sensitive to a high-fat diet, which can lead to accumulation of lipid and glucose and arterial wall damage, thus resulting in coronary artery stenosis and subsequent CHD-like symptoms.^22^^,^^23^

In this study, a high-fat diet feeding method was employed to generate a CHD model, and a moderate aerobic exercise intervention was given to a group of the CHD rats. Aerobic exercise resulted in an increase in LVEF% and LVFS%, a decrease in LVIDd and LVIDs, improved cardiac function, reduced myocardial damage, and improved glucose and lipid metabolism. Wang et al.^24^ reported that moderate aerobic exercise in humans and animals is capable of reducing triglycerides and fasting blood glucose concentrations, decreasing the resistance to insulin, as well as modulating the glucolipid balance. Considerable evidence indicates that excessively released inflammatory factors and inflammatory reactions are crucial factors affecting the occurrence and development of cardiovascular disease. Myocardial injury will activate the inflammatory response in the body, and release abundant inflammatory factors, which in turn contribute to the process of myocardial injury, fibrosis, and repair.^25^^,^^26^ The authors found that the concentrations of IL-6, TNF- and IL-1 and in the myocardial tissue of CHD rats were increased. Aerobic exercise intervention not only reduced IL-6, IL-1 and TNF- in myocardial tissue, but also increased IL-10 concentrations.

NF-B and IB form a trimer and exist in the cytoplasm. Under the action of external stimuli or inflammatory factors, the body can degrade IB and release NF-B. NF-B can be promptly transferred to the nucleus, thus modulating the expression of various influencing factors and cell adhesion factors, as well as producing a cascading amplification influence.^27^^,^^28^ The activated NF-κB signaling pathway depends on the activation of NF-κB and the phosphorylation of p38. Increased p38 phosphorylation level can promote the activation of NF-κB and transfer into the nucleus to regulate STAT3 phosphorylation, which mediates the activation of inflammation and apoptosis.^29^ IB- is the earliest discovered IB molecule and one of the inhibitory proteins of NF-Κb.^30^ In this study, NF-B mRNA level in cardiomyocytes of CHD rats was notedly increased, whereas IB- mRNA level was reduced. These results indicate that aerobic exercise can suppress the NF-B signaling pathway in cardiomyocytes of CHD rats, thereby restraining the release of inflammatory factors.

In conclusion, this study shows that aerobic exercise can not only reduce myocardial damage, release of inflammatory factors and NF-B level, but also improve cardiac function and glucolipid metabolism in CHD rats. Furthermore, the findings are consistent with a mechanism connected to the inhibition of the NF-B signaling pathway. The above conclusions promisingly provide a theoretical and experimental basis for clinical treatment and rehabilitation programs for CHD patients.

## Declaration of competing interest

The authors declare no conflicts of interest.
